# Web-Based Versus Usual Care and Other Formats of Decision Aids to Support Prostate Cancer Screening Decisions: Systematic Review and Meta-Analysis

**DOI:** 10.2196/jmir.9070

**Published:** 2018-06-26

**Authors:** Sofia Baptista, Elvira Teles Sampaio, Bruno Heleno, Luís Filipe Azevedo, Carlos Martins

**Affiliations:** ^1^ Department of Community Medicine, Information and Health Decision Sciences Faculty of Medicine University of Porto Porto Portugal; ^2^ Serpa Pinto Family Health Unit Agrupamento de Centros de Saúde Porto Ocidental Porto Portugal; ^3^ Chronic Diseases Research Centre NOVA Medical School NOVA University of Lisbon Lisboa Portugal; ^4^ Centre for Health Technology and Services Research University of Porto Porto Portugal

**Keywords:** decision making, decision aid, internet, patient participation, prostate, screening

## Abstract

**Background:**

Prostate cancer is a leading cause of cancer among men. Because screening for prostate cancer is a controversial issue, many experts in the field have defended the use of shared decision making using validated decision aids, which can be presented in different formats (eg, written, multimedia, Web). Recent studies have concluded that decision aids improve knowledge and reduce decisional conflict.

**Objective:**

This meta-analysis aimed to investigate the impact of using Web-based decision aids to support men’s prostate cancer screening decisions in comparison with usual care and other formats of decision aids.

**Methods:**

We searched PubMed, CINAHL, PsycINFO, and Cochrane CENTRAL databases up to November 2016. This search identified randomized controlled trials, which assessed Web-based decision aids for men making a prostate cancer screening decision and reported quality of decision-making outcomes. Two reviewers independently screened citations for inclusion criteria, extracted data, and assessed risk of bias. Using a random-effects model, meta-analyses were conducted pooling results using mean differences (MD), standardized mean differences (SMD), and relative risks (RR).

**Results:**

Of 2406 unique citations, 7 randomized controlled trials met the inclusion criteria. For risk of bias, selective outcome reporting and participant/personnel blinding were mostly rated as unclear due to inadequate reporting. Based on seven items, two studies had high risk of bias for one item. Compared to usual care, Web-based decision aids increased knowledge (SMD 0.46; 95% CI 0.18-0.75), reduced decisional conflict (MD –7.07%; 95% CI –9.44 to –4.71), and reduced the practitioner control role in the decision-making process (RR 0.50; 95% CI 0.31-0.81). Web-based decision aids compared to printed decision aids yielded no differences in knowledge, decisional conflict, and participation in decision or screening behaviors. Compared to video decision aids, Web-based decision aids showed lower average knowledge scores (SMD –0.50; 95% CI –0.88 to –0.12) and a slight decrease in prostate-specific antigen screening (RR 1.12; 95% CI 1.01-1.25).

**Conclusions:**

According to this analysis, Web-based decision aids performed similarly to alternative formats (ie, printed, video) for the assessed decision-quality outcomes. The low cost, readiness, availability, and anonymity of the Web can be an advantage for increasing access to decision aids that support prostate cancer screening decisions among men.

## Introduction

### Prostate Cancer and Screening

According to the GLOBOCAN worldwide estimates of cancer incidence and mortality produced by the International Agency for Research on Cancer, there were 1,111,700 new cases of prostate cancer and 307,500 prostate cancer deaths in 2012, making prostate cancer the second most commonly diagnosed cancer in men and the fifth leading cause of cancer deaths among men [1].

Screening for prostate cancer remains a controversial issue, particularly after data from two major trials were released. The United States Prostate*,* Lung, Colorectal, and Ovarian Cancer Screening Trial found no benefits from using prostate-specific antigen (PSA) screening for prostate cancer diagnoses [2]. The results from the European Randomised Study of Screening for Prostate Cancer with data truncated at 13 years concluded that one prostate cancer death would be avoided and 27 excess cases detected per 781 men invited for screening with PSA [3]. Overdiagnosis was estimated to be as high as 41%. The proportion of prostate cancer that would never have led to clinical symptoms resulted in unnecessary biopsy procedures and treatment with potential side effects, which may include urinary, sexual (eg, erectile dysfunction), and gastrointestinal complications [4]. The CAP Randomized Clinical Trial was recently published and reported no significant difference in prostate cancer mortality with PSA screening after a median follow-up of 10 years but an increase in the detection of low-risk prostate cancer cases [5].

Although often encouraged by media and health care providers, prostate cancer screening is currently recommended by only a few organizations. After reviewing the available evidence, the United States Preventive Services Task Force released a draft recommendation in 2017, assigning a “C” grade recommendation to prostate cancer screening in men 55-69 years old, stating that the potential benefits and adverse effects of PSA-based screening are closely balanced in that age group. The decision about whether to be screened should be an individual one based on conversations with the physician about the benefits and adverse effects of screening in order to help men make a decision based on personal values and preferences [6]. Many experts defend a shared decision-making process involving doctor and patient, using validated decision aids. In fact, many guidelines issued by medical organizations such as the European Association of Urology [7], the American Cancer Society [8], and the American College of Physicians [9] support a shared decision-making process for prostate cancer screening.

### Shared Decision Making and Decision Aids

According to the International Patient Decision Aids Standards Collaboration (IPDAS), decision aids are evidence-based tools designed to help people participate in decision making about health care options with the aim of improving the quality of the decision. Many study groups have focused on the development of decision aids to support shared decision making [10,11].

As established in the original IPDAS background document, two constructs are critical for establishing the effectiveness of a decision aid: (1) the quality of both the decision-making process and (2) the actual decision. For the quality of the decision-making process, five attributes are defined, all of which are measured by different scales: (1) recognizing that a decision needs to be made, (2) feeling informed about the options, (3) understanding what values matter most for the decision, (4) discussing preferences with their practitioner, and (5) being involved in decision making. Concerning the decision quality, two core attributes should be measured: (1) a patient’s knowledge of the options and outcomes and (2) agreement between the chosen option and the features that matter most for the patient [12].

The most recent systematic review and meta-analysis assessing the impact of decision aids for screening decisions concluded that decision aids can increase patient knowledge, make people feel clearer about their values, reduce decisional conflict, and promote an active patient role in decision making [13]. The authors state that more studies are needed to deepen understanding of format issues such as Web-based delivery of decision aids. In addition, if new studies can be included in the systematic review, it may be possible to sort out the reasons for heterogeneity of results (eg, the format of the decision aid). Another systematic review, focusing on decision aids for prostate cancer screening, reported similar results [14].

Decision aids may be implemented in different formats, including written (eg, pamphlet/booklet), multimedia (eg, video, DVD), or Web-based. Syrowatka et al, in a systematic review and meta-analysis that assessed computer-based decision aids for any preference-sensitive medical decision, concluded that decision aids are associated with a significant improvement in knowledge and decrease in decisional conflict. However, results were limited by high levels of heterogeneity [15]. Nevertheless, the scope of the latter review was broader, including any preference-sensitive medical decision. Thus, it did not specifically address prostate cancer screening. In addition, the authors included all decision aid formats that could be accessed with a computer (eg, Web-based, videobooklet, CD-ROM). With the increasing use and ease of access to the internet, the Web has been proposed as a promising way of delivering decision aids. Therefore, it is important to assess the impact of Web-based decision aids in the prostate cancer screening decision-making process, but the number of studies addressing this subject to date have been scarce and showed mixed results.

The IPDAS Collaboration identified 12 dimensions to assess quality of patient decision aids, one of which focused on the delivery of decision aids on the internet [16]. In fact, several theories point out the potential benefits of the internet to provide broad long-term dissemination of information that can be targeted and tailored to patient needs and preferences. Hence, IPDAS emphasized that a comprehensive systematic review focusing on the internet delivery of decision aids was needed [17].

To our knowledge, ours is the first systematic review and meta-analysis to compare Web-based decision aids with usual care and other formats of decision aids. We sought to investigate their impact on decision quality for men making a screening decision regarding prostate cancer.

## Methods

### Criteria for Considering Studies for This Review

We included randomized controlled trials (RCTs) involving men who had not been previously diagnosed with prostate cancer and who were making screening decisions concerning prostate cancer. We included studies comparing Web-based decision aids to several parameters: (1) no intervention/usual care or (2) alternative decision aids formats. For interventions to be considered Web-based, they had to correspond to any program accessed over a network connection using HTTP or through a Web-based app. According to this definition, materials such as CD-ROMs or DVDs, although computer-based, were not considered Web-based. Thus, studies with such interventions were excluded. We included studies in which at least one quality of decision-making outcome (eg, knowledge, decisional conflict, and involvement in decision making) was reported. Screening behavior, either the intention to undergo PSA screening or undergoing the actual PSA screening, were defined as secondary outcomes.

### Search Methods for Identification of Studies

#### Electronic Searches

Our search strategy for this review included searching electronic medical and social science databases: (1) PubMed, (2) Cumulative Index to Nursing and Allied Health Literature (CINAHL), (3) PsycINFO, and (4) Cochrane CENTRAL (Cochrane Central Register of Controlled Trials). Whenever possible, the search strategies (Multimedia Appendix 1) used a combination of free text and database-specific subject headings. The search was conducted in November 2016.

#### Searching Other Resources

We also searched trial registries (World Health Organization, National Institutes of Health, ClinicalTrials.gov), reference lists of included trials, and the Decision Aid Library Inventory.

### Data Collection and Analysis

#### Selection of Studies

Two reviewers screened the titles and abstracts of all retrieved articles after employing the search strategy. Those included after screening were accessed in full text. Authors were contacted to clarify study eligibility. Disagreements were resolved by consensus among 3 reviewers.

### Data Extraction and Management

Data extraction was performed independently by 2 reviewers. Extracted data included study design and setting, numbers, and other characteristics of study participants and interventions in addition to outcomes and other information thought to be relevant. Whenever different publications reported on the same trial, the data corresponding to the latest follow-up were included. For cluster RCTs, we collected effect estimates and standard errors from analyses that took the clustering into account. Study authors were contacted when more detailed information was needed. Disagreements were resolved by consensus. When available, data resulting from imputation were used in the analysis in accordance with an intention-to-treat approach.

### Assessment of Risk of Bias in Included Studies

Assessment of risk of bias was performed using the Cochrane tool for judging risk of bias [18].

### Measures of Treatment Effect and Data Synthesis

We used mean differences (MD) for continuous variables that were measured with the same instrument, standardized mean differences (SMD) when a similar outcome was assessed with different instruments, and relative risks (RR) for dichotomous variables. Continuous variables were standardized to a scale of 0-100. In cases where outcome data (eg, standard deviations) were missing, we tried to reach one of the study authors by email to request the complete measures. If we were unsuccessful in obtaining the data from authors, we derived standard deviations from standard errors or confidence intervals [18].

We analyzed studies comparing Web-based decision aids to usual care separately from studies comparing Web-based decision aids to decision aids presented in a diverse format. Review Manager 5.3.5 software was used to estimate meta-analytical-weighted treatment effects across studies [19]. Data analysis was conducted with a random-effects model given the heterogeneity among studies being pooled.

### Sensitivity Analysis

A sensitivity analysis was conducted in order to reassess the effect measures after excluding trials classified as having high risk of bias for any of the chosen parameters and after applying the fixed-effects model. For the knowledge outcome, a sensitivity analysis was done using MDs as an alternative to SMDs. Although we opted for SMD to pool knowledge across studies since different constructs were used to measure this outcome, the use of MD could also be defensible, as the scale itself is the same.

## Results

### Results of the Search and Description of Studies

**Figure 1:**
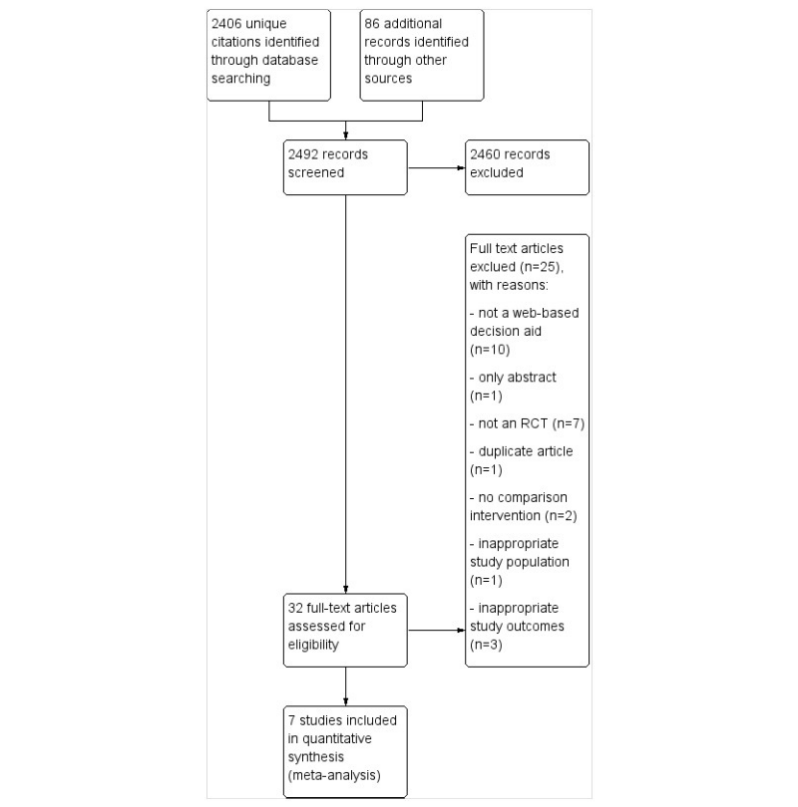
Study flow diagram. RCT: randomized controlled trial.

The electronic database search retrieved 2406 unique citations (2536 records), and 86 additional citations were identified through other sources (Figure 1). Of the 32 full-text articles assessed, 25 were excluded (Figure 1 describes the reasons, and further details are provided in Multimedia Appendix 2). We contacted 4 investigators to clarify methodological issues and to complete the extracted data when necessary.

### Included Studies

Seven studies met our inclusion criteria and were included in the meta-analysis. The characteristics of included studies are presented in Table 1.

The studies were published between 2003 and 2013; five were based in the United States [20-26], one in Australia [25], and one in the United Kingdom [26]. A total of 4714 men with ages ranging from 45-75 years participated in the seven included studies. Five of the studies recruited men from a primary care setting [20-22,24,26], one through a radio and newspaper advertisement [25], and another from an industrial manufacturing worksite [23]. Six of the studies randomized individual patients [21,22,24-26], while one used the worksite as the unit of randomization [23]. For this review, we referred to control interventions as usual care unless they fulfill the deﬁnition of a patient decision aid. Among the included studies, five studies compared Web-based decision aid performance to the usual care [22-24,26], four studies compared Web-based decision aids to printed decision aids [20,24-26], and two studies compared Web-based decision aids to video decision aids [20,25]. In addition to containing information about the options and outcomes regarding prostate cancer screening, all decision aids used in the studies provided a values clarification tool, except for one [25]. All included studies assessed knowledge, and six measured decisional conflict [21-26]. Others reported outcomes included anxiety [25,26], satisfaction with decision [20,24], decision-making role [21,23,25], and intention to undergo and actually undergoing PSA screening [21-26]. All studies used a parallel design, except Allen et al, who used a cluster RCT. Allen et al used a generalized estimating equations analysis and thus properly accounting for the cluster design and the possible associated unit-of-analysis errors.

**Table 1:** Characteristics of included studies.

Study	Methods	Participants	Comparison	Outcomes
Frosch 2003 [20]	RCT^a^, 2 groups: video decision versus Web-based decision aids	Men aged ≥50 years considering PSA^b^ screening in a preventive medicine clinic (USA): 112 (Web)/ 114 (video)	Decision aid: Web-based, with information, pros and cons of PSA testing, experiences of other patients, values clarification exercise Active comparator: Video, same content of the Web decision aid	Primary outcome measures: (1) participant ratings of convenience, effort required, and satisfaction with the intervention, (2) knowledge about prostate cancer screening and treatment, and (3) choice of undergoing PSA test
Krist 2007 [21]	RCT, comparing Web-based decision and paper-based decision aids versus no intervention (usual care)	Men aged 50-70 years considering PSA screening in a primary care setting (USA): 226 (Web)/ 196 (paper)/ 75 (usual care-control)	Decision aid: Web-based information about prostate cancer, screening, screening benefits, and known risks, current uncertainties. The website was reviewed by a general decision aid expert and several content experts. Active comparator: print brochure, which duplicated the content of the website Comparator: usual care	Primary outcome: patient-reported control preferences scale score. Other: Prostate cancer screening knowledge, time spent discussing screening, topics covered in the discussion, decisional conflict scale score and whether a PSA test was ordered
Frosch 2008 [22]	RCT, 4 groups: Web-based decision aid (1) vs Web decision aid + chronic disease trajectory (2) vs chronic disease trajectory (3) vs usual care (internet info) (4)	Men aged ≥50 years considering PSA screening in a preventive medicine clinic (USA): 155 (1) + 152 (2) + 153 (3) + 151 (4)	Decision aid: information about prostate cancer screening and treatment, with physician and patient testimonials contrasting different preferences and decisions Active comparator: chronic disease trajectory model that prompted patients to express utilities for outcomes associated with a prostate cancer life course by contrasting screening with no screening in its impact on quality of life and longevity Comparator: links to public websites on prostate cancer screening maintained by the American Cancer Society and the Centers for Disease Control and Prevention	Primary outcome measures: (1) knowledge; (2) actual option; (3) decisional conflict. Other outcomes: (1) treatment preference if cancer diagnosed and (2) concern about prostate cancer
Allen 2010 [23]	RCT, 2 groups (Web and control)	Men aged ≥45 years considering PSA screening (USA): 398 (Web)/ 414 (no intervention)	Decision aid: Web-based (content based on expert opinion and guidelines from IPDAS^c^). Comparator: no intervention	Primary outcomes: (1) decisional status, (2) prostate cancer knowledge, (3) decision self-efficacy, (4) consistency between values, and (5) screening decision. Secondary outcomes: (1) preference for control in decision making and (2) decisional conflict
Taylor 2013 [24]	RCT, 3 groups (Web, paper, and control)	Men aged 45-70 years considering PSA screening in a primary care setting (USA): 631 (Web)/ 630 (paper)/ 632 (usual care)	Decision aid: Web-based Active comparator: printed decision aid Comparator: usual care Both decision aids share same content: (1) description of screening tests and possible results, (2) information about treatment options, risks and adverse effects, (3) a review of prostate cancer risk factors and encouragement to discuss screening with a physician, and (4) a 10-item values clarification tool; and resources for more information. Web-based decision aids also included interactive features (eg, testimonials, interactive values clarification tools)	Knowledge, decisional conflict scale, satisfaction with decision scale, prostate cancer screening uptake. Measured at baseline, and then after 1 and 13 months
Ilic 2008 [25]	RCT, 3 groups (Web, paper, and video)	Men aged ≥45 years considering PSA screening in Australia, recruited by radio and newspaper advertisements: 56 (Web)/ 50 (pamphlet)/ 55 (video)	Decision aid: Web-based Active comparator: pamphlet Active comparator: video Decision aid contents: (1) epidemiology on prostate cancer, (2) diagnostic process, (3) treatment options, and (4) the associated benefits/risks	Primary outcome: decisional conflict. Secondary outcomes: (1) knowledge and (2) anxiety, consumer decision-making role and screening interest
Evans 2010 [26]	RCT, 4 groups: 2 intervention groups (Web- and paper-based) and 2 control groups (questionnaire and usual care)	Men aged 50-75 years considering PSA screening in a primary care setting (UK): 129 (Web)/ 126 (paper)/127 (questionnaire)/ 132 (usual care)	Decision aid: Web-based - Prosdex: information, pros and cons of PSA testing, other patient experiences, values clarification exercise Active comparator: paper version with the text of the website Active comparator: questionnaire Comparator: usual care	Primary outcome: knowledge of prostate cancer and PSA. Other: attitudes towards PSA testing; behavior (intention to undergo PSA testing), anxiety, decisional conflict, and actually undergoing of PSA test (at 6 months)

^a^RCT: randomized controlled trial.

^b^PSA: prostate-specific antigen.

^c^IPDAS: International Patient Decision Aids Standards.

### Risk of Bias in Included Studies

Assessments of the risk of bias for each study are summarized in Multimedia Appendix 3 and the authors’ support for each judgment are presented in Multimedia Appendix 4. Random sequence generation was rated as being at low risk of bias in most of the studies (6/7, 86%) and unclear risk of bias in one study. Allocation concealment was considered low risk of bias in five studies (5/7, 71%) and unclear risk in the remaining two studies.

Blinding of participants and personnel was assessed as being at low risk of bias in one study (1/7, 14%), unclear risk of bias in four studies (4/7, 57%), and high risk in two studies (2/7, 29%) [20,21]. All studies were evaluated as being a low risk of bias regarding blinding of outcome assessment. All studies were rated as low risk of attrition bias that relates to incomplete outcome data. Five studies (5/7, 71%) were classified as unclear risk of bias regarding selective reporting due to the lack of information about public registration of the trial protocol. The other two studies had a registered protocol and were rated as low risk of bias for the selective reporting parameter. When assessing other sources of bias, six studies were rated as low risk of bias (6/7, 86%). The remaining study was considered unclear risk of bias as study groups were not similar in size [20].

### Effects of Interventions

The summary of the findings is found in Multimedia Appendix 5.

### Knowledge

All seven studies assessed patient knowledge in the meta-analysis. Studies tested knowledge through questionnaires based on the content of the decision aids. The number of correct answers was transformed into a scale ranging from 0% (no correct answers) to 100% (all correct answers).

#### Web-Based Decision Aids Versus Usual Care

**Figure 2:**
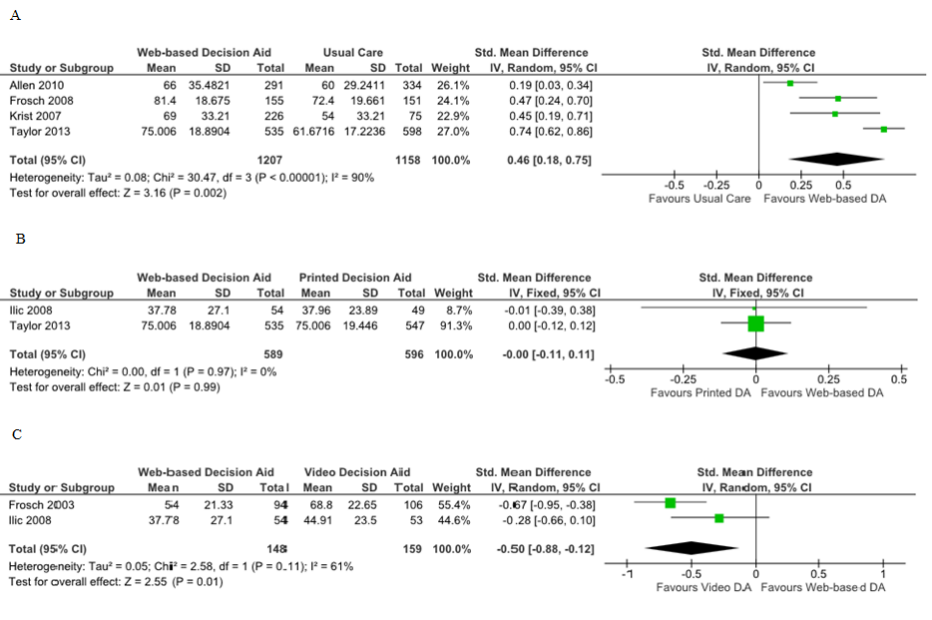
Forest plots of standardized mean differences for knowledge. A) Web-based decision aids (DA) versus usual care, B) Web-based decision aids versus printed decision aids, C) Web-based decision versus video decision aids.

Four studies included knowledge comparisons for this outcome in the meta-analysis. One study used a different way for grading the questionnaire (1 point for a correct answer, 0 for any unanswered item, and –1 for an incorrect answer), so data could not be transformed for the scale described above. In addition, no standard deviations could be obtained; thus data could not be pooled [26]. Compared to the usual care, patients allocated to Web-based decision aids had higher average knowledge scores (SMD 0.46; 95% CI 0.18-0.75; Figure 2). The study that was not included in the meta-analysis showed a higher statistically signiﬁcant average score for the Web-based decision aid group in comparison with the usual care. Four of the five RCTs assessing knowledge for Web-based decision aids compared to usual care demonstrated a statistically signiﬁcant improvement in knowledge in the Web-based decision aid group [19-24]. Taylor et al reassessed knowledge at 13 months, and the Web-based decision aid group continued to register a statistically significant increase in median scores compared to the usual care group [25].

#### Web-Based Versus Printed Decision Aids

Four studies assessed knowledge for the comparison of Web-based to printed decision aids, but only data from two studies could be pooled. The scale used by Evans et al was not convertible to 0%-100% scale [24]. Additionally, standard deviations for study results could not be obtained for the Evans et al and Krist et al studies; these studies did not find any differences between groups regarding this outcome. No differences in the average knowledge scores were found for this comparison (SMD 0.00; 95% CI –0.11 to 0.11; Figure 2) [21,26].

#### Web-Based Versus Video Aids

With regard to the comparison of Web-based decision to video decision aids, the Web-based group registered lower average knowledge scores (SMD –0.50; 95% CI –0.88 to –0.12; Figure 2) for the pooled data for two studies. Frosch et al found a smaller nonstatistically significant difference between the two groups when only the participants who reviewed the complete set of materials were considered for analysis [22].

### Decisional Conflict

Six of the seven studies measured patient decisional conflict using the decisional conflict scale [20-25]. The decisional conflict scale consists of five subscales, and total scores range from 0 (no decisional conflict) to 100 (extremely high decisional conflict). When comparing Web-based aids to usual care or alternative formats of decision aids, a negative score corresponds to a reduction in decisional conflicts, which favors Web-based decision aids.

#### Web-Based Decision Aids Versus Usual Care

**Figure 3:**
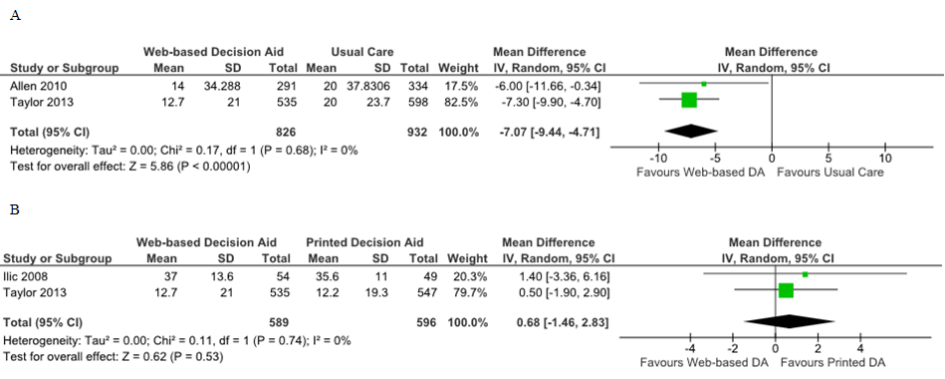
Forest plots of mean differences for decisional conflict. A) Web-based decision aids (DA) versus usual care, B) Web-based decision aids versus printed decision aids.

Five studies compared Web-based decision aids to usual care in terms of decisional conflict [22,23,26]. It was not possible to pool data from two studies due to lack of standard deviation of the results [21,26]. Krist et al did not find a significant difference between the two groups in contrast with the findings of Evans et al who reported a significant higher decisional conflict for the usual care group [21,26]. Frosch et al reported the results using subscales without providing standard deviation data; this study finding showed significantly higher decisional conflict for the usual care group in the subscales of “feeling informed” and “support in decision making”, and no difference was found for the subscales “uncertainty” and “having made an effective decision” [22]. The overall MD for decisional conflict comparing Web-based decision aids versus usual care was –7.07% (95% CI –9.44 to –4.71; Figure 3).

#### Web-Based Decision Versus Printed Decision Aids

Four studies assessed decisional conflict by comparing Web-based decision to printed decision aids [21,24-26]. The MD for pooled data from two studies was 0.68 (95% CI –1.46 to 2.83; Figure 3). Data from the Evans et al and Krist et al studies could not be included for meta-analysis because standard deviations could not be obtained [21,26]. Reported mean scores for decisional conflicts were similar for the two groups in the Krist et al study [21]. Evans et al did not find any statistically significant differences for decisional conflict when a Web-based decision aid was compared to a printed one [26].

#### Web-Based Decision Versus Video Decision Aids

Ilic et al did not find any statistical differences regarding mean decisional conflict scores for patients when exposed to Web-based decision compared to video decision aids [25].

### Participation in Decision Making

Three of the seven studies evaluated participation in decision making using the Control Preferences Scale (CPS) [21,23,25], which consists of five statements (A to E), two of which reflect patient controlled decision making, another one refers to shared decision making, and the last two reflect practitioner-controlled decision making. Decision aids are intended to enhance a patient’s active role in decision making. Therefore, a pooled RR >1 for group differences in CPS statements A to C favors Web-based decision aids, and a pooled RR <1 for statements D and E also favors Web-based decision aids.

#### Web-Based Decision Aids Versus Usual Care

**Figure 4:**
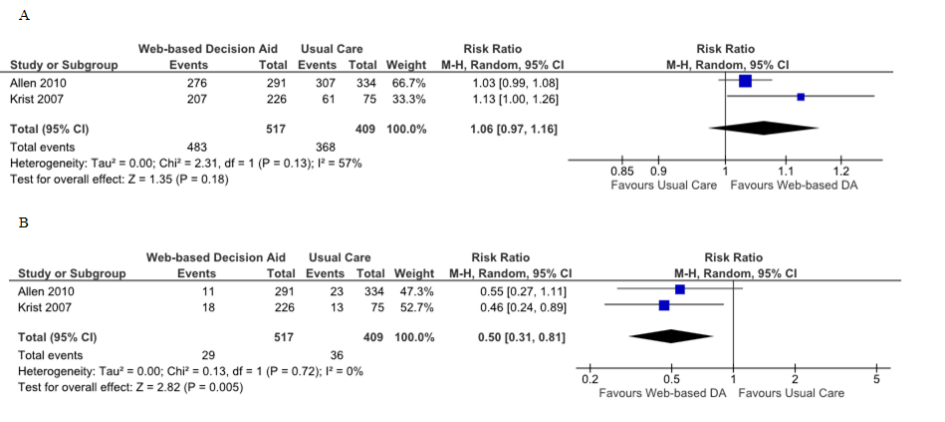
Forest plots of relative risks for participation in decision making. A) Patient controlled or shared decision making: Web-based decision aid (DA) versus usual care, B) Practitioner controlled decision making: Web-based decision aid versus usual care.

When comparing Web-based decision aids to usual care in terms of a patient-controlled or active role in the decision-making process, the pooled RR was 1.06 (95% CI 0.97-1.16; Figure 4). For the practitioner-controlled role, a pooled RR of 0.50 was obtained, which compared Web-based decision aids to usual care (95% CI 0.31-0.81; Figure 4).

#### Web-Based Decision Versus Printed Decision Aids

**Figure 5:**
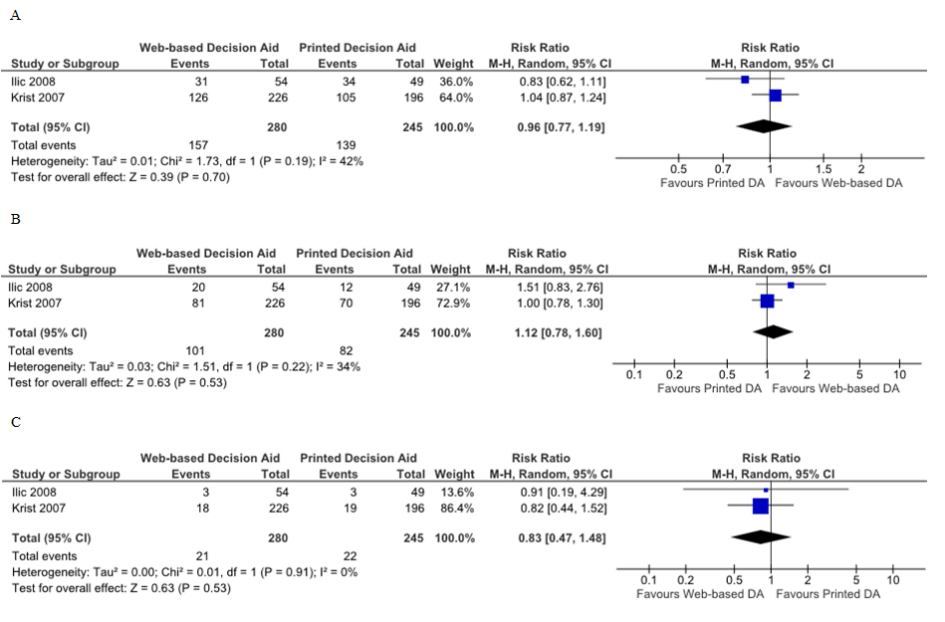
Forest plot of relative risks for participation in decision making: Web-based decision aids versus printed decision aids (DA). A) Patient controlled, B) Shared decision making, C) Practitioner controlled.

Regarding patients who assumed an active role according to the CPS, the pooled RR was 0.96 (95% CI 0.77-1.19; Figure 5). The pooled RR for the same comparison for a collaborative role in decision making was 1.12 (95% CI 0.78-1.60; Figure 5). Finally, when pooling data that compared Web-based decision to printed decision aids in terms of a passive role according to the CPS, the RR obtained was 0.83 (95% CI 0.47-1.48; Figure 5).

#### Web-Based Decision Versus Video Decision Aids

Ilic et al was the only study assessing participation in decisions comparing Web-based decision to video decision aids. No statistically significant differences between groups were found for active (RR 0.89; 95% CI 0.66-1.21), collaborative (RR 1.15; 95% CI 0.68-1.95), or passive patient role in decision making according to the CPS (RR 1.47; 95% CI 0.26-8.46) [25].

### Screening Behavior: Preferred Option

Three studies investigated the preferred patient options concerning prostate screening using the PSA test [20,22,23]; Evans et al and Ilic et al evaluated answers using a 5-point Likert-like response scale [25,26]. Allen et al reported agreement with the statement “want to be screened” [23].

#### Web-Based Decision Aids Versus Usual Care

**Figure 6:**
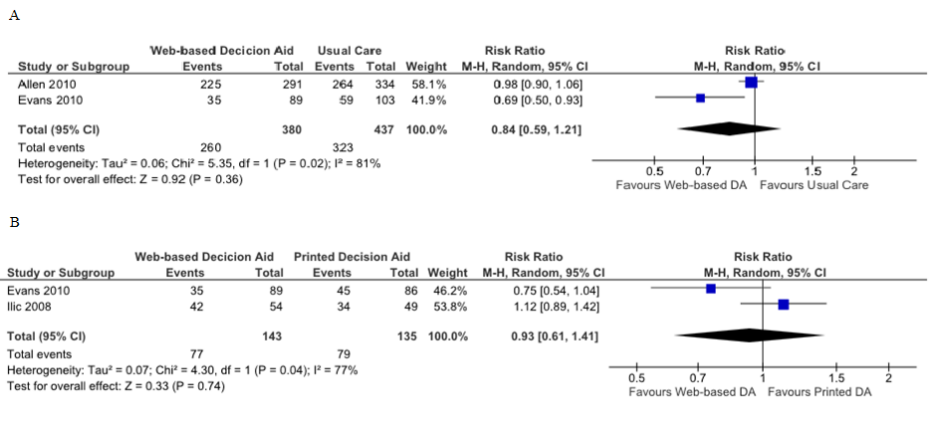
Forest plot of relative risks for screening behavior - preference for prostate-specific antigen test. A) Web-based decision aids (DA) versus usual care, B) Web-based decision aids versus printed decision aids.

The pooled RR for two studies comparing the preference for having a PSA test for patients using a Web-based decision aid in comparison to usual care was 0.84 (95% CI 0.59-1.21; Figure 6).

#### Web-Based Decision Versus Printed Decision Aids

When comparing Web-based decision to printed decision aids, the overall pooled RR indicating a preference for PSA screening was 0.93 (95% CI 0.61-1.41; Figure 6).

#### Web-Based Decision Versus Video Decision Aids

Ilic et al assessed the preference for the PSA screening test with a 5-point response scale. When comparing those who responded either “definitely want” and “probably want” in the Web-based decision aid and video groups, there was no significant difference (RR 1.29; 95% CI 0.99-1.67) [25].

### Screening Behavior: PSA Test

Using different methods, five studies investigated the actual choice of PSA screening: (1) Evans et al asked general practitioners to review participant’s medical records [26], (2) Frosch et al also relied on medical records [22], (3) Taylor et al assessed patients’ self-reported PSA screening at 13 months [24], (4) Krist et al used patients’ reports of PSA tests ordered [19], and (5) Frosch et al searched for PSA test requests in medical records [20].

#### Web-Based Decision Aids Versus Usual Care

When comparing Web-based decision aids to usual care groups after pooling data for screening uptake, the obtained RR was 1.0 (95% CI 0.89-1.11; Multimedia Appendix 6).

#### Web-Based Decision Aids Versus Printed Decision Aids

The overall pooled RR was 1.04 (95% CI 0.97-1.12; Multimedia Appendix 6) when comparing PSA tests in patients exposed to Web-based decision aids to patients receiving printed decision aids.

#### Web-Based Decision Aids Versus Video Decision Aids

The only study assessing PSA test uptake in patients receiving a Web-based decision aid versus patients receiving a video decision aid revealed a slight difference for the comparison (RR 1.12; 95% CI 1.0-1.25) [20].

### Sensitivity Analysis

We investigated the potential bias resulting from including studies that were assessed as high risk of bias for any of the seven criteria considered, which resulted in exclusion of two studies for this analysis [20,21]. Most results remained similar, with the following exception: the differences in knowledge between Web-based decision and video groups became nonsignificant. The difference for a practitioner-controlled role in decision making between Web-based decision aids and usual care also became nonsignificant. After applying a fixed-effect model, the results were also compared to the results retrieved in the first analysis with the random-effect model. Results were similar for all outcomes and comparisons, with one exception. When comparing Web-based decision aids to usual care for a patient active or collaborative role in decision, with a slight decimal change, the difference became significant. These similar results of fixed-effect model to random-effect model diminish risk of bias due to “small study effects” (ie, the potential of the included small studies to overestimate effect sizes).

Taking into account IPDAS criteria [11], decision aids should offer a values clarification tool. After a verification process, which included contact with authors, Ilic et al was the only one of the included studies that did not contain such tool. We conducted the analysis after removal of this study. The results were similar to those obtained when the study was included.

When using mean differences instead of standardized mean differences for knowledge, results were similar for the comparisons of Web-based decision aids versus usual care (MD 10.66%; CI 95% 6.78-14.53), Web-based decision versus printed decision aids (MD –0.01%; CI 95% –2.23 to 2.22), and Web-based decision versus video decision aids (MD –11.9%; CI 95% –19.19 to –4.61).

### Heterogeneity

Statistically significant heterogeneity was found for knowledge when comparing Web-based decision aids to usual care.

## Discussion

### Principal Findings and Evidence

In comparison with usual care, Web-based decision aids significantly increased knowledge, reduced decisional conflict, and reduced the practitioner-controlled role in the decision-making process. No differences were found regarding patients assuming an active or collaborative role in decision making or in terms of screening behavior. When comparing Web-based decision with printed decision aids, no differences were found for knowledge, decisional conflict, participation in decision, or screening behavior. Compared to video decision aids, Web-based decision aids showed lower average knowledge scores and a slight decrease in PSA screening uptake, while no differences were found regarding participation in decision making. None of the studies assessed decisional conflict for these comparisons.

There is high-quality evidence that suggests that Web-based decision aids when compared to printed decision aids perform similarly in improving men’s knowledge regarding prostate cancer screening and reducing decisional conﬂict. There is moderate-quality evidence that Web-based decision compared to printed decision aids show no differences in screening behavior. There is also low-quality evidence that Web-based decision aids resulted in lower knowledge scores when compared with video decision aids.

### Interpretation the Context of Existing Literature

Our results are similar to those from other systematic reviews and meta-analyses indicating the superiority of decision aids (in any format) in comparison with usual care, but to our knowledge, our study is the first to compare Web-based decision aids to alternative formats in the context of prostate cancer screening decisions. Stacey et al, in a Cochrane meta-analysis of decision aids for people facing screening or treatment issues, found decision aids to improve people’s knowledge, reduce decisional conflict, promote an active patient role in decision making, and reduce the number of patients choosing to undergo PSA screening [13]. In a systematic review and meta-analysis of features of computer-based decision aids for any preference-sensitive medical decision, Syrowatka et al indicated that decision aids are associated with significant knowledge improvement and decrease in decisional conflict. However, results were limited by high levels of heterogeneity [15]. Ilic et al, in a systematic review assessing the effectiveness of decision aids for decision making in prostate cancer testing, also reported a reduction in decisional conflict and a statistically significant improvement in knowledge [14].

Syrowatka et al reported that computer-based decision aids were associated with significant improvements in knowledge and decisional conflict compared to usual care or alternative aids [15]. On the other hand, our results show Web-based decision aids perform similarly to printed decision aids in terms of decision quality outcomes. Comparisons should be made with caution, since the systematic review by Syrowatka et al addressed all computer-based decision aids (not only Web-based) and any preference-sensitive medical decision. It may not be surprising that Web-based and printed decision aids perform similarly in the context of a trial, since the contents of the decision aid in both arms is the same. Therefore, the current evidence supports the use of decision aids. However, either decision aid format may be used depending on individual patient’s preference. In addition, we hypothesize that in a busy day-to-day clinical practice, with limited time to talk to patients, Web-based decision aids may have greater potential, allowing patients to easily access and review the material prior to the encounter with the physician, which may impact the shared decision-making process.

Only two studies compared Web-based to video decision aids, which limits our conclusions, particularly concerning the fact that Web-based decision aids showed lower average knowledge scores [20,25]. However, we can hypothesize that older men making a prostate cancer screening decision may not be very familiar with internet use. Of note, in Frosch et al, the video decision aid population arm had a specifically allocated time to watch the video, which was different from the Web-based decision aid group. This difference may have increased adherence to video visualization. This study showed that the video group had significantly more probability of reviewing the materials, while in the Web-based decision aid group, only 53.5% watched the entire presentation, and 39.5% had not reviewed any part of it. In fact, the authors reported that for those in the Web-based decision aid group who reviewed the entire presentation, knowledge scores were similar to those from the video group [20]. More studies are needed in order to address the comparison of decision aids reviewed at an assigned versus self-allocated time.

### Strengths and Limitations

Among the included studies, the risk of bias was higher for the blinding of participants and personnel criteria. Post-hoc analysis removing studies at high risk of bias yielded the same results, except that no differences were found for knowledge comparing Web-based to video decision aids and no differences for a practitioner-controlled role in decision making when comparing Web-based decision aids to usual care.

Several limitations must be considered while analyzing our conclusions. For most outcomes, the number of studies was low, making it difficult to assess for publication bias. The different contents of the decision aids of each study also limited their comparison. We tried to pool data only when the same scale or procedure was used to evaluate each outcome. However, different ways of formulating the questions may also pose a limitation to our conclusions. The way studies measured screening behavior could also have introduced bias. The methods used, such as self-reported screening, review of medical records, and evaluation of intention to undergo screening, may not be reliable. PSA screening decisions may have to be made by men several times in their life, so lack of long-term follow-up in the included studies also limits the understanding of the impact of Web-based decision aids over time. Taylor et al was the study with the longest follow-up (13 months) [24].

Some studies mention visualization rates of the decision aids. Allen et al refers rates from 23%-59% in the intervention Web-based decision aid group [23]. In Frosch et al, 86.7% of the Web-based decision aid arm participants at least clicked on the link provided [22]. It is possible that if all men effectively viewed the materials, the impact of decision aids could be amplified. Results could also have been influenced due to the fact that people in the printed decision aid arms could have the decision aid with them while answering the questionnaires.

Finally, participants in the majority of included studies were white men with high educational levels, which limits the generalization of results for other populations, such as low literacy men and cultural minorities. Allen et al addressed a low literacy population in nonclinical settings (worksites) that differed from the other included studies [23]. Taylor et al also included many participants from a low socioeconomic background [24]. More trials should focus on these populations and investigate the delivery of decision aids in different settings.

We hypothesize that the statistically significant heterogeneity found for knowledge when comparing Web-based decision aids and usual care in conjunction with Web-based to video decision aids may be due to the fact that the tests used for this assessment in the included studies were not standardized.

### Conclusions

According to this analysis, the Web format seems to have a similar effect to printed or video decision aids in terms of increase in knowledge and decrease in decisional conflict. This provides evidence to use decision aids to support a patient’s prostate cancer screening decision, whether it is a Web-based decision aid or alternative format like a printed or video version. Of note, most included studies were published 7 or more years ago. In the last several years, the internet has become even more ubiquitous and easy to use with many public places providing it at no cost, which may be an important feature to increase access to decision aids. Another potential advantage, especially for health preference sensitive issues, is the anonymity that Web allows. Increasingly more decision aids will likely become available through this media, and more men will be skilled enough to search for them online. More RCTs are needed to further compare the impact of these alternative decision aid formats in decision making and to analyze their influence not only in the short term, but also over time. In addition, more studies are needed to deepen our understanding of the unique features of Web-based decision aids, such as virtual connectivity, interactivity, tailoring, as well as to compare Web-based decision aids with video and printed decision aids in terms of implementation and dissemination strategies and cost-effectiveness analyses [17].

## Appendix

Multimedia Appendix 1 Search strategies for electronic databases.

Multimedia Appendix 2 Characteristics of excluded studies.

Multimedia Appendix 3 Risk of bias graph and risk of bias summary.

Multimedia Appendix 4 Characteristics of included studies.

Multimedia Appendix 5 Summary of findings.

Multimedia Appendix 6 Forest plots of RRs for screening behavior - PSA test uptake.

## Abbreviations

CENTRAL: Cochrane Central Register of Controlled Trials
CINAHL: Cumulative Index to Nursing and Allied Health Literature
CPS: Controlled Preferences Scale
IPDAS: International Patient Decision Aids Standards Collaboration
MD: mean difference
PSA: prostate specific antigen
RCT: randomized controlled trial
RR: relative risk
SMD: standardized mean difference
