# Supplementary material for: Web-Based Versus Usual Care and Other Formats of Decision Aids to Support Prostate Cancer Screening Decisions: Systematic Review and Meta-Analysis
Source: J Med Internet Res. 2018 Jun 26;20(6):e228. doi: 10.2196/jmir.9070 (PMC6043730; doi:10.2196/jmir.9070)
Supplement: Multimedia Appendix 1 [file jmir_v20i6e228_app1.pdf]

Multimedia Appendix 1. Search strategies for electronic databases.

Medline(Pubmed)

#	Searches	
1	Prostatic Neoplasms [mh]	
2	prostate cancer*[tw]OR prostate carcinoma* [tw]OR prostate malignan*[tw] OR prostate tumor*[tw] OR prostate tumour*[tw] OR prostate neoplasm*[tw] OR prostate adeno*[tw]	
3	#1 OR #2	
4	randomizedcontrolled trial [pt]	
5	controlledclinical trial [pt]	
6	randomized [tiab]	
7	placebo [tiab]	
8	groups [tiab]	
9	drug therapy [sh]	
10	randomly [tiab]	
11	trial [tiab]	
12	#4 OR #5 OR #6 OR #7 OR #8 OR #9 OR #10 OR #11	
13	animals [mh] not (humans [mh] and animals [mh])	
14	#12 NOT #13	
15	decisionsupporttechniques[mh]	
16	decision support systems, clinical [mh]	
17	decisiontrees[mh]	
18	decision making [mh] OR choice behavior [mh]	
19	audiovisual aids[mh]	
20	decision* aid* [tw]OR decision* support*[tw]OR decision* tool*[tw] OR decision* instrument* [tw]OR decision* technolog[tw]OR decision* technique* [tw]OR decision* system*[tw]OR decision* program* [tw]OR decision* algorithm* [tw]OR decision* process* [tw]OR decision*method* [tw]OR decision* intervention* [tw] OR decision* material*[tw]	
21	risk communication tool* [tw]OR risk assessment tool* [tw]OR risk information tool* [tw]OR risk communication method* [tw]OR risk assessment method* [tw]OR risk information method*[tw]	
22	shared decision making[tw]	
23	informed choice*[tw]	
24	informed decision*[tw]	
25	patient decision*[tw]	
26	#14 OR #15 OR #16 OR #17 OR #18 OR #19 OR #20 OR #21 OR #22 OR #23 OR #24 OR #25	
27	decision Making, Computer-Assisted [mh]	
28	medical informatics [mh]	
29	user-computer interface [mh]	

30	Internet [mh]	
31	Computers[mh]	
32	web based[tw]	
33	computer*[tw]	
34	interactive[tw]	
35	#27 OR #28 OR #29 OR #30 OR #31 OR #32 OR #33	
36	#3 AND #14 AND #26 AND #35	

CENTRAL

Description: limited to trials

#	Searches	
1	MeSH descriptor: [Prostatic Neoplasms] explode all trees	
2	prostate near cancer*	
3	prostate near neoplasm*	
4	prostate near carcinoma*	
5	prostate near tumour*	
6	prostatenear tumor*	
7	prostatenearadeno*	
8	#1 OR #2 OR #3 OR #4 OR #5 OR #6 OR #7	
9	MeSH descriptor: [Decision Support Techniques] Single MeSH term (unexploded)	
10	MeSH descriptor: [Decision Support Systems, Clinical] Single MeSH term (unexploded)	
11	MeSH descriptor: [decision trees] Single MeSH term (unexploded)	
12	MeSH descriptor: [decision making] Single MeSH term (unexploded)	
13	MeSH descriptor: [choice behavior] Single MeSH term (unexploded)	
14	MeSH descriptor: [audiovisual aids] Single MeSH term (unexploded)	
15	(decision* NEAR/3 support*) OR (decision* NEAR/3 aid*) OR (decision* NEAR/3 tool*) OR (decision* NEAR/3 instrument*) OR (decision* NEAR/3 technolog*) OR (decision* NEAR/3 technique*) OR (decision* NEAR/3 system*) OR (decision* NEAR/3 program*) OR (decision* NEAR/3 algorithm*) OR (decision* NEAR/3 process*) OR (decision* NEAR/3 method*) OR (decision* NEAR/3 intervention*) OR (decision* NEAR/3 material*) : ti,ab,kw	
16	(risk communication" NEAR/3 tool*) or ("risk communication" NEAR/3 method*) or ("risk information" NEAR/3 tool*) or ("risk information" NEAR/3 method*) or ("risk assessment" NEAR/3 tool*) or ("risk assessment" NEAR/3 method*): ti,ab,kw	
17	shared decision making: ti,ab,kw	
18	informed choice*: ti,ab,kw	
19	informed decision*: ti,ab,kw	
20	patient decision*: ti,ab,kw	
21	#9 OR #10 OR #11 OR #12 OR #13 OR #14 OR #15 OR #16 OR #17 OR #18 OR #19 OR #20	
22	MeSH descriptor: [decision Making, Computer-Assisted] Single MeSH term (unexploded)	
23	MeSH descriptor: [medical informatics] Single MeSH term (unexploded)	
24	MeSH descriptor: [user-computer interface] SingleMeSH term (unexploded)	

25	MeSH descriptor: [Internet] Single MeSH term (unexploded)	
26	MeSH descriptor: [Computers] Single MeSH term (unexploded)	
27	web based: ti,ab,kw	
28	computer*: ti,ab,kw	
29	interactive: ti,ab,kw	
30	#21 OR #22 OR #23 OR #24OR #25 OR #26 OR #27 OR #28 OR #29	
31	#8 AND #21 AND #30	
32	#31 in trials	

CINAHL (via EBSCO)

#	Searches	
S1	(MH "Prostatic Neoplasms+"): Search modes - Boolean/Phrase	
S2	(TX “prostate N6 (cancer* OR carcinoma* OR malignan* or tumor* OR tumour* OR neoplasm* OR adeno*)”): Search modes - Boolean/Phrase	
S3	S1 OR S2: Search modes - Boolean/Phrase	
S4	AB (random* or trial or placebo*): Search modes - Boolean/Phrase	
S5	TI (random* or trial or placebo*): Search modes - Boolean/Phrase	
S6	(MH “Quantitative Studies”) : Search modes - Boolean/Phrase	
S7	(MH “Placebos”) : Search modes - Boolean/Phrase	
S8	(MH “Random assignment”) : Search modes - Boolean/Phrase	
S9	(MH “Clinical Trials+”) : Search modes - Boolean/Phrase	
S10	(PT “Clinical Trial”) : Search modes - Boolean/Phrase	
S11	(PT “randomi#ed controlled trial”) : Search modes - Boolean/Phrase	
S12	S4 OR S5 OR S6 OR S7 OR S8 OR S9 OR S10 OR S11: Search modes - Boolean/Phrase	
S13	(MH "decision support techniques+"): Search modes - Boolean/Phrase	
S14	(MH "decision support systems, clinical"): Search modes - Boolean/Phrase	
S15	(MH "decision trees"): Search modes - Boolean/Phrase	
S16	(MH "decision making"): Search modes - Boolean/Phrase	
S17	(MH "educational technology"): Search modes - Boolean/Phrase	
S18	(decision* N3 support*) or (decision* N3 aid*) or (decision* N3 tool*) or (decision* N3 instrument*) or (decision* N3 technolog*) or (decision* N3 technique*) or (decision* N3system*) or (decision* N3 program*) or (decision* N3 algorithm*) or (decision* N3 process*) or (decision* N3method*) or (decision* N3 intervention*) or (decision* N3 material*) : Search modes - Boolean/Phrase	
S19	(“risk communication” N3 tool*) or (“risk communication” N3 method*) or (“risk information” N3 tool*) or (“risk information” N3 method*) or (“risk assessment” N3 tool*) or (“risk assessment” N3 method*): Search modes - Boolean/Phrase	
S20	“shared decision making”: Search modes - Boolean/Phrase	
S21	“informed choice*”: Search modes - Boolean/Phrase	
S22	“informed decision*”: Search modes - Boolean/Phrase	
S23	“patient decision*”: Search modes - Boolean/Phrase	
S24	S13 OR S14 OR S15 OR S16 OR S17 OR S18 OR S19 OR S20 OR S21 OR S22 OR S23	
S25	(MH "decision Making, Computer Assisted +"): Search modes - Boolean/Phrase	
S26	(MH "medical informatics"): Search modes - Boolean/Phrase	
S27	(MH “user-computer interface+"): Search modes - Boolean/Phrase	
S28	(MH "Internet"): Search modes - Boolean/Phrase	
S29	computer* N1 decision making:Search modes - Boolean/Phrase	
S30	“web based”: Search modes - Boolean/Phrase	
S31	“computer*”: Search modes - Boolean/Phrase	
S32	“interactive”: Search modes - Boolean/Phrase	

S33	S25 OR S26 OR S27 OR S28 OR S29 OR S30 OR S31 OR S32	
S34	S3 AND S12 AND S24 AND S33	

PsycInfo (via EBSCO)

#	Searches	
S1	(MA "Prostatic Neoplasms+"): Search modes - Boolean/Phrase	
S2	(TX “prostate N6 (cancer* OR carcinoma* OR malignan* or tumor* ORtumour* OR neoplasm* OR adeno*)”): Search modes - Boolean/Phrase	
S3	S1 OR S2: Search modes - Boolean/Phrase	
S4	AB (random* or trial or placebo*): Search modes - Boolean/Phrase	
S5	TI (random* or trial or placebo*): Search modes - Boolean/Phrase	
S6	(TX “control*”) : Search modes - Boolean/Phrase	
S7	(MA “Placebos”) : Search modes - Boolean/Phrase	
S8	(MA “Clinical Trials+”) : Search modes - Boolean/Phrase	
S9	S4 OR S5 OR S6 OR S7 OR S8: Search modes - Boolean/Phrase	
S10	(MA " decision support techniques+"): Search modes - Boolean/Phrase	
S11	(MA " decision support systems, clinical+ "): Search modes - Boolean/Phrase	
S12	(MA " decision trees"): Search modes - Boolean/Phrase	
S13	(MA "decision making"): Search modes - Boolean/Phrase	
S14	(MA " choice behavior"): Search modes - Boolean/Phrase	
S15	(MA "audiovsual aids"): Search modes - Boolean/Phrase	
S16	(decision* N3 support*) or (decision* N3 aid*) or (decision* N3 tool*) or (decision* N3 instrument*) or (decision* N3 technolog*) or (decision* N3 technique*) or (decision* N3 system*) or (decision* N3 program*) or (decision* N3 algorithm*) or (decision* N3 process*) or (decision* N3method*) or (decision* N3 intervention*) or (decision* N3 material*) : Search modes - Boolean/Phrase	
S17	(“risk communication” N3 tool*) or (“risk communication” N3 method*) or (“risk information” N3 tool*) or (“risk information” N3 method*) or (“risk assessment” N3 tool*) or (“risk assessment” N3 method*):Search modes - Boolean/Phrase	
S18	“shared decision making”: Search modes - Boolean/Phrase	
S19	“informed choice*”: Search modes - Boolean/Phrase	
S20	“informed decision*”: Search modes - Boolean/Phrase	
S21	“patient decision*”: Search modes - Boolean/Phrase	
S22	S10 OR S11 OR S12 OR S13 OR S14 OR S15 OR S16 OR S17 OR S18 OR S19 OR S20 OR S21	
S23	(MA "decision Making, Computer Assisted +"): Search modes - Boolean/Phrase	
S24	(MA "medical informatics +"): Search modes - Boolean/Phrase	
S25	(MA "user-computer interface+"): Search modes - Boolean/Phrase	
S26	(MA "Internet+"): Search modes - Boolean/Phrase	
S27	(MA " Computers+"): Search modes - Boolean/Phrase	

S28	computer* N1 decision making:	
S29	“web based”: Search modes - Boolean/Phrase	
S30	“computer*”: Search modes - Boolean/Phrase	
S31	“interactive”: Search modes - Boolean/Phrase	
S32	S23 oR S24 OR S25 OR S26 OR S27 OR S28 OR S29 OR S30 OR S31	
S33	S3 AND S9 AND S22 AND S32	
