# Supplementary material for: Web-Based Versus Usual Care and Other Formats of Decision Aids to Support Prostate Cancer Screening Decisions: Systematic Review and Meta-Analysis
Source: J Med Internet Res. 2018 Jun 26;20(6):e228. doi: 10.2196/jmir.9070 (PMC6043730; doi:10.2196/jmir.9070)
Supplement: Multimedia Appendix 2 [file jmir_v20i6e228_app2.pdf]

Multimedia Appendix 2. Characteristics of Excluded Studies.

	Study	Reason for Exclusion
1	Partin 2004	Not a web-based intervention<br>“pamphlet subjects were<br>mailed an educational pamphlet developed by the study<br>team, and video subjects were mailed the educational video<br>evaluated in previous studies”<br>“Roughly<br>90% of the patients in our study returned the videos as<br>requested in preaddressed, postage-paid envelopes.”
2	Witteman H 2008<br><br>A recommender system for prostate cancer websites.	E-mail requesting full-text sent to holly.witteman@fmed.ulaval.ca(5/1/2017)<br>Answer received on 9/1/2017 – the work was not developed further than the existing abstract
3	Landrey 2013	Not a web-based intervention<br>Daniel.Matlock@ucdenver.edu – email sent 5/1/2017<br>Answer 5/1/2017 – intervention sent via traditional mail
4	Watson 2006	Not a web-based intervention<br>“letters”, “mailed copy” of pamphlet
5	Chan 2011	Not a web-based intervention<br><br>“usinga script and slides with video clips of role models to trigger discussion. Materials included Men’s Guides, short booklets that presented information in the same sequence as the slides and videoclips.
6	Dorfman 2010	Innapropriate study design – not a RCT<br>Feasilbility study for printed and web-based DA
7	Driscoll 2008	Innapropriate study design – community interventions, not a RCT<br>Not a patient DA<br>Not a web-based intervention
8	Ellison 2008	Innapropriate study design – quasiexperimentalpostinterventional, not a RCT<br>Compares 2 web-based DA (enhanced vs usual)
9	Kerns 2008	Compares involvement of resident vs faculty physicians rather than web-based to other format of DA, thus does not fulfill the outcomes in the inclusion criteria<br>“outcomes were compared for resident<br>physicians (both second- and third-year residents) andfaculty physicians”<br>Innapropriatedtudy population
10	Layton 2011	Innapropriate study design (quasiexperimental, not RCT)
11	Lewis 2015	Intervention computer-based but not web-based (DVD)
12	Myers 2005	Not a web-based DA in comparison (booklet vs enhanced intervention)
13	Myers 2010	Not a web-based DA in comparison (booklet vs enhanced intervention with decision counseling session with a nurse)
14	Owens 2014	Innapropriate study design – not a RCT (convenience sample)
15	Owens 2015	Innapropriate study design – not a RCT (convenience sample); duplicate
16	Rubel 2010	Not a web-based DA (booklet)
17	Salkeld 2013	Abstract of article (Salkeld 2016) in a book of abstracts; duplicate
18	Salkeld 2016	Both DA in comparison web based (fixed attributes version vs personalized)

19	Sheinfeld 2007	Not RCT (descriptive usability test of a web-based DA)
20	Volk 2008 influence	Not a web-based DA
21	Watts 2013	Both DA web-based (tailored vs non-tailored)
22	Wilkes	Inadequate outcomes
23	Volk 2008	CD-ROM, not a web-based DA
24	Starosta 2015	Inadequate outcomes
25	Tomko 2015	Inadequate outcomes
