# Supplementary material for: Web-Based Versus Usual Care and Other Formats of Decision Aids to Support Prostate Cancer Screening Decisions: Systematic Review and Meta-Analysis
Source: J Med Internet Res. 2018 Jun 26;20(6):e228. doi: 10.2196/jmir.9070 (PMC6043730; doi:10.2196/jmir.9070)
Supplement: Multimedia Appendix 4 [file jmir_v20i6e228_app4.pdf]

Multimedia Appendix 4. Characteristics of included studies (by study ID).

**Author / Year**	**Evans 2010**
Methods	Randomised controlled trial, composed of 4 groups: 2 intervention groups (web-based DA / paper DA) and 2 control groups
Participants	Men considering PSA screening in a Primary Care setting in Wales (UK), aged 50 to 75 years: 129 (web) / 126 (paper) / 127 (questionnaire-control 1) / 132 (usual care-control 2)
Interventions	DA: Web-based PSA DA Prosdex: information, pros and cons of PSA testing, experiences of other patients, values clarification exercise.<br>Compare: Paper version: paper document comprising the text of the website.<br>Compare: received a questionnaire<br>Compare: received nothing (usual care).
Outcomes	Knowledge of prostate cancer and PSA, the primary outcome of the study; attitude toward PSA testing; behavior, using the proxy measure of intention to undergo PSA testing. (p.4) Anxiety (six-item short form of the Spielberger State Anxiety Inventory) and decisional conflict (Decisional Conflict Scale) Actual uptake of the PSA test (at 6 months). (p.5)
**Risk of Bias**	Author's judgement / Support for judgement
Random sequence generation (selection bias)	**Unclear risk** – "A staff member from each practice generated a list of potential participants (…)A random sample of 100 men was selected from the list. (…)The process ensured individual level randomization to one of two intervention groups or to one of two control groups (p.3)
Allocation concealment (selection bias)	**Low risk** - Affirmative consent forms from each practice were transferred to the research officer (author NJ-W) who allocated each participant with a number provided remotely by the trial statistician (author RN) to ensure concealment. (p.3)
Incomplete outcome data (attrition bias)<br>All outcomes	**Low risk** – Figure 1 (flow diagram); table 1 (baseline characteristics of participants). "Outcomes were compared between groups on an "intention to treat" basis." (p. 5)
Selective reporting (reporting bias)	**Low risk –** the trial was registered
Other bias	**Low risk** – there is no evidence of other sources of bias
Blinding of participants and personnel<br>(performance bias)<br>All outcomes	**Unclear –** not specified in the text
Blinding of outcome assessment (detection bias)<br>All outcomes	**Low risk –** although unclear blinding of outcome assessment, outcomes were objectively measured

**Author / Year**	**Frosch 2003**
Methods	Randomised controlled trial, composed of 2 groups: video DA vs. web-based DA
Participants	Men aged >= 50 years considering PSA screening in a preventive Medicine clinic in San Diego (USA): 112 (web) / 114 (video)
Interventions	DA: Web-based PSA DA: information, pros and cons of PSA testing, experiences of other patients, values clarification exercise.<br>Compare: Video same content of the web decision aid
Outcomes	Primary outcome measures: (1) participant ratings of convenience, effort required, and satisfaction with the intervention; (2) knowledge about prostate cancer screening and treatment; (3) choice of PSA test. (p.782)
**Risk of Bias**	Author's judgement / Support for judgement
Random sequence generation (selection bias)	**Low Risk** – Once the questionnaire was completed, the server automatically assigned the participant to 1 of the 2 groups based on a sequence previously generated with a random number generator. (p.782)
Allocation concealment (selection bias)	**Unclear risk –** Not explicitly mentioned, but implied – "All men were provided with a unique arbitrary ID and password, allowing<br>them to access a secure internet site containing all study materials.(…) Participants assigned to the internet group were unable to view the video without this being known to the investigators." (p.782)
Incomplete outcome data (attrition bias)<br>All outcomes	**Low risk** – Figure 1 (flow diagram); table 1 (baseline characteristics of participants, p. 784). "In examining participants' PSA knowledge, we therefore used an *Intention-to-Treat* analytic approach" (p. 782) "Consistent with an Intention-to-Treat analytic approach, data for the PSA knowledge variable were imputed differentially for those participants who did not complete<br>follow-up assessments." (p.783)
Selective reporting (reporting bias)	**Unclear –** no information provided
Other bias	**Low risk** – there is no evidence of other sources of bias
Blinding of participants and personnel<br>(performance bias)<br>All outcomes	**High risk –** No blinding of study personnel assured, which could influence decision making process and PSA test uptake. **"**Participants assigned to the video group were informed that they could view the video at HAC 30 minutes prior to their appointment." (p.782)
Blinding of outcome assessment (detection bias)<br>All outcomes	**Low risk –** although unclear blinding of outcome assessment, outcomes were objectively measured.

**Author / Year**	**Frosch 2008**
Methods	Randomised controlled trial, composed of 4 groups: web-based DA vs. web DA + chronic disease trajectory vs. chronic disease trajectory vs. usual care (internet info)
Participants	Men aged >= 50 years considering PSA screening in a preventive Medicine clinic in San Diego (USA): 155 + 152 + 153 + 151
Interventions	DA: traditional didactic decision aid that provided comprehensive information about prostate cancer screening and treatment and included physician and patient testimonials contrasting different preferences and decisions.<br>COMPARE: chronic disease trajectory model that activated patients to express utilities for outcomes associated with a prostate cancer life course by contrasting screening with no screening in its impact on quality of life and longevity<br>Usual care: links to public Web sites on prostate cancer screening maintained by the American Cancer Society and the Centers for Disease Control and Prevention.
Outcomes	Primary outcome measures: knowledge, actual option, decisional conflict. Other outcomes: treatment preference if cancer diagnosed, concern about prostate cancer (p.)
**Risk of Bias**	Author's judgement / Support for judgement
Random sequence generation (selection bias)	**Low Risk** – "A computer algorithm then randomly assigned partici- pants to the 4 study groups." (p.364)
Allocation concealment (selection bias)	**Low Risk** – allocation was revealed after participants had completed the baseline questionnaire and signed consent.
Incomplete outcome data (attrition bias)<br>All outcomes	**Low risk** – Figure 1 (flowchart, p. 365); table 1 (baseline characteristics of participants, p. 366).<br>Intention to treat analysis (p. 364).
Selective reporting (reporting bias)	**Unclear –** no information provided
Other bias	**Low risk** – there is no evidence of other sources of bias.
Blinding of participants and personnel (performance bias)<br>All outcomes	**Unclear -** blinding of participants but unclear blinding of personnel.
Blinding of outcome assessment (detection bias)<br>All outcomes	**Low risk –** although unclear blinding of outcome assessment, outcomes were objectively measured.

**Author / Year**	**Krist 2007**
Methods	Randomised controlled trial, comparing web-based DA and paper-based DA vs. no intervention (usual care)
Participants	Men aged 50 to 70 years considering PSA screening in a primary care setting in Virginia (USA): 226 (web) / 196 (paper) / 75 (usual care-control)
Interventions	DA: Web-based: information about prostate cancer, screening concepts, potential screening benefits, and known risks, as well as current uncertainties. The Web site was reviewed by a general decision aid expert and several content experts.<br>Compare: print brochure: duplicated the content of the website.
Outcomes	Primary outcome was the patient-reported Control Preferences Scale (CPS) score (p. 113)<br>Prostate cancer screening knowledge, time spent discussing screening, topics covered in the discussion, Decisional Conflict Scale (DCS) score and whether a PSA test was ordered. (p. 114)
**Risk of Bias**	Author's judgement / Support for judgement
Random sequence generation (selection bias)	**Low risk** – "coordinator referred to pre-generated randomization tables to inform the participant to which arm he was randomized" (p.3)
Allocation concealment (selection bias)	**Low risk** – "At the time of enrollment, the allocation was concealed from the coordinator. (p.113)
Incomplete outcome data (attrition bias)<br>All outcomes	**Low risk** – Figure 1 (flow diagram); table 1 (characteristics of the study population). "Comparisons between groups were made on an intention-to-treat basis." (p. 114)
Selective reporting (reporting bias)	**Unclear –** no information provided
Other bias	**Unclear** – groups not similar in size, but apparently no evidence of other biases
Blinding of participants and personnel (performance bias)<br>All outcomes	**High risk –** physicians not blinded, which could affect the decision making and screening uptake. "Although allocation was concealed, we made no attempts to blind physicians to the patient's group." (p. 113)
Blinding of outcome assessment (detection bias)<br>All outcomes	**Low risk –** although unclear blinding of outcome assessment, outcomes were objectively measured.

**Author / Year**	**Taylor 2013**
Methods	Randomised controlled trial
Participants	Men aged 45 to 70 years considering PSA screening in a primary care setting in Washigton DC (USA): 631 (web) / 630 (paper) / 632 (usual care)
Interventions	DA: web-based DA<br>Compare: printed DA<br>Compare: usual care<br>Both DA share same content: " introductory material about the prostate gland; a description of screening tests and possible results; information about treatment options, risks, and adverse effects; a review of PCa risk factors and encouragement to discuss screening with a physician; a 10-item values clarification tool; and resources for more information". (p.4) Web-based DA adds interactive features (eg. Testemonials, interactive values clarification tool,…)
Outcomes	Knowledge, Decisional conflict scale, Satisfaction with decision scale, prostate cancer screening uptake<br>Measured at baseline, 1 month and 13 months
**Risk of Bias**	**Author's judgement / Support for judgement**
Random sequence generation (selection bias)	**Low risk** – "the interviewer used a computer-generated random allocation sequence to assign participants in a 1:1:1 ratio to the web DA, print DA, or UC. Randomization was stratified by site and self-reported race. (pp.3-4)
Allocation concealment (selection bias)	**Low risk** - "the interviewer used a computer-generated random allocation sequence to assign participants in a 1:1:1 ratio to the web DA, print DA, or UC. Randomization was stratified by site and self-reported race. (pp.3-4)
Incomplete outcome data (attrition bias)<br>All outcomes	**Low risk** – Figure flow diagram (p.14); table 1 (baseline characteristics of participants (p.15). "(…) assessed longitudinal effects on 1-month and 13-month outcomes using intention-to-treat analyses." (p. 5)
Selective reporting (reporting bias)	**Low risk –** the trial was registered
Other bias	**Low risk** – there is no evidence of other sources of bias
Blinding of participants and personnel<br>(performance bias)<br>All outcomes	**Unclear –** not specified in the text if the interviewers knew the study arm of the participants while doing the 1 and 13 months assessments; nevertheless, interviewers did not conduct the outcome assessments.
Blinding of outcome assessment (detection bias)<br>All outcomes	**Low risk –** although unclear blinding of outcome assessment, outcomes were objectively measured

**Author / Year**	**Ilic 2008**
Methods	Randomised controlled trial
Participants	Men aged >= 45 years considering PSA screening in Australia, recruited by radio and newspaper advertisement:56 (web) / 50 (pamphlet) / 55 (video)
Interventions	DA: web-based DA<br>Compare: pamphlet<br>Compare: video<br>“Australian epidemiology on prostate cancer, the diagnostic process, treatment options, and the benefits/risks associated with the “prostate journey”.” (p.2)
Outcomes	Primary outcome: decisional conflict. Secondary outcomes: knowledge, anxiety, Consumer Decision-Making Role and screening interest.
**Risk of Bias**	**Author’s judgement / Support for judgement**
Random sequence generation (selection<br>bias)	**Low risk** – “allocation sequence was generated using a random number generator by the trial statistician” (p.2)
Allocation concealment (selection bias)	**Low risk** – “To randomize a participant, the lead investigator would enter the participant’s ID, initials, age, and last 3 digits of their land and/or mobile number. The application would then return the group to which the participant had been allocated. (p.2)
Incomplete outcome data (attrition bias)<br>All outcomes	**Low risk** – Figure 1- participant flow (p.3); table 1 - baseline demographic details of participants (p.4).
Selective reporting (reporting bias)	**Unclear –** no information provided
Other bias	**Low risk** – there is no evidence of other sources of bias
Blinding of participants and personnel<br>(performance bias)<br>All outcomes	**Low risk** - **“**Details were collected by telephone at baseline and at 1-week post intervention by a single outcome assessor (KE) who was blinded to group allocation.” (p.2)
Blinding of outcome assessment (detection<br>bias)<br>All outcomes	**Low risk – “**Details were collected by telephone at baseline and at 1-week post intervention by a single outcome assessor (KE) who was blinded to group allocation.” (p.2)

**Author / Year**	**Allen 2010**
Methods	Randomised controlled trial
Participants	Men aged >= 45 years considering PSA screening in the USA:398 (web) / 414 (no intervention)
Interventions	DA: web-based DA<br>Compare: no intervention<br>"Content of the DA was based on expert opinion regarding information necessary for informed decision making (10, 19), as well as guidelines from the International Patient Decision Aid Standards (IPDAS)(….)" (p.3)
Outcomes	Primary outcome: decisional status, prostate cancer knowledge, decision self-efficacy, consistency between values and screening decision. Secondary outcomes: preference for control in decision making; decisional conflict.
**Risk of Bias**	**Author's judgement / Support for judgement**
Random sequence generation (selection bias)	**Low risk** – "Sites were blocked on size (total employees <500; ≥500) and percent of male employees (<50%; ≥50%), and randomly assigned by computer-generated random numbers to condition within blocks." (p.3)
Allocation concealment (selection bias)	**Unclear risk** – not specifically addressed in the article
Incomplete outcome data (attrition bias)<br>All outcomes	**Low risk** – Figure 1- participant flow (p.17); table 1 - baseline demographic details of participants (p.18).
Selective reporting (reporting bias)	**Unclear –** no information provided regarding protocol
Other bias	**Low risk** – there is no evidence of other sources of bias
Blinding of participants and personnel<br>(performance bias)<br>All outcomes	**Unclear risk** - not specifically addressed in the article
Blinding of outcome assessment (detection bias)<br>All outcomes	**Low risk –** although unclear blinding of outcome assessment, outcomes were objectively measured
