# Supplementary material for: Web-Based Versus Usual Care and Other Formats of Decision Aids to Support Prostate Cancer Screening Decisions: Systematic Review and Meta-Analysis
Source: J Med Internet Res. 2018 Jun 26;20(6):e228. doi: 10.2196/jmir.9070 (PMC6043730; doi:10.2196/jmir.9070)
Supplement: Multimedia Appendix 5 [file jmir_v20i6e228_app5.pdf]

Multimedia Appendix 5. Summary of Findings.

**Patient web-based decision aids compared with other format decision aids for adults considering prostate cancer screening decision**						
**Patient or population:** adults considering prostate cancer screening decision<br>**Settings:** all settings<br>**Intervention:** web-based decision aid<br>**Comparison:** other format decision aids						
**Outcomes**	**Illustrative comparative benefits* (95% CI)**		**Relative or Absolute Effect Measures (95% CI)**	**Number of participants (studies)**	**Quality of the evidence (GRADE)**	Comments
	**Assumed Benefit**	**Corresponding benefit**				
	**Other format decision aid**	**Web-based decision aid**				
**Knowledge: web decision aid versus printed decision aid** [standardized to a 0-100 score scale] [soon after exposure to decision aid]	The mean knowledge score was 56.48% (ranging from 37.96% to 75.00%)	The mean knowledge score was 56.39% (ranging from 37.78% to 75.00%)	SMD 0.00 [95% CI -0.11, 0.11]	1185 (2 studies)	⊕⊕⊕⊕ **high**[1]	
**Knowledge: web decision aid versus video decision aid** [standardized to a 0-100 score scale] [soon after exposure to decision aid]	The mean knowledge score was 56.86% (ranging from 44.91% to 68.80%)	The mean knowledge score was 56.39% (ranging from 37.78% to 75.00%)	SMD -0.50 [95% CI -0.88, -0.12]	307 (2 studies)	⊕⊕⊝⊝ **low**[2,3]	

**Decisional Conflict: web decision aid versus printed decision aid** [Decisional Conflict Scale, standardized to a 0 to 100 score] [soon after exposure to decision aid]	The mean DCS score was 23.9 (ranging from 12.2 to 35.6)	The mean DCS score was 24.85 (ranging from 12.7 to 37.0)	MD 0.68 [95% CI -1.46, 2.83]	1185 (2 studies)	⊕⊕⊕⊕ **high**[1]
**Participation in decision making: web decision aid versus printed decision aid – practitioner controlled decision making** [soon after physician's appointment]	**89 patients per 1000**	**74 patients per 1000**	RR 0.83 [95% CI 0.47, 1.48]	525 (2 studies)	⊕⊕⊝⊝ **low**[2,3]
**Participation in decision making: web decision aid versus printed decision aid – shared decision making** [soon after physician's appointment]	**334 patients per 1000**	**374 patients per 1000**	RR 1.12 [95% CI 0.78, 1.60]	525 (2 studies)	⊕⊕⊝⊝ **low**[2,4]
**Screening Behaviour - PSA test uptake: web decision aid versus printed decision aid** [up to 13 months after physician's appointment]	**500 patients per 1000**	**520 patients per 1000**	RR 1.04 [95% CI 0.97, 1.12]	1477 (3 studies)	⊕⊕⊕⊝ **moderate**[2]

*The basis for the **assumed risk** (e.g. the median control group risk across studies) is provided in footnotes. The **corresponding risk** (and its 95% confidence interval) is based on the assumed risk in the comparison group and the **relative effect** of the intervention (and its 95% CI).
CI: Confidence interval; **SMD:** Standardized Mean Difference; **MD:** Mean Difference; **RR:** Risk Ratio.

GRADE Working Group grades of evidence
High quality: Further research is very unlikely to change our confidence in the estimate of effect.
Moderate quality: Further research is likely to have an important impact on our confidence in the estimate of effect and may change the estimate.
Low quality: Further research is very likely to have an important impact on our confidence in the estimate of effect and is likely to change the estimate.
Very low quality: We are very uncertain about the estimate.

1. None of the studies measuring this outcome were at high risk of bias.
2. The GRADE rating was downgraded given the risk of bias.
3. The GRADE rating was downgraded given the lack of precision.
4. The GRADE rating was downgraded given the lack of consistency.
5. The GRADE rating was downgraded given the lack of directness.
