# Supplementary figures and images for: Web-Based Versus Usual Care and Other Formats of Decision Aids to Support Prostate Cancer Screening Decisions: Systematic Review and Meta-Analysis
Source: J Med Internet Res. 2018 Jun 26;20(6):e228. doi: 10.2196/jmir.9070 (PMC6043730; doi:10.2196/jmir.9070)

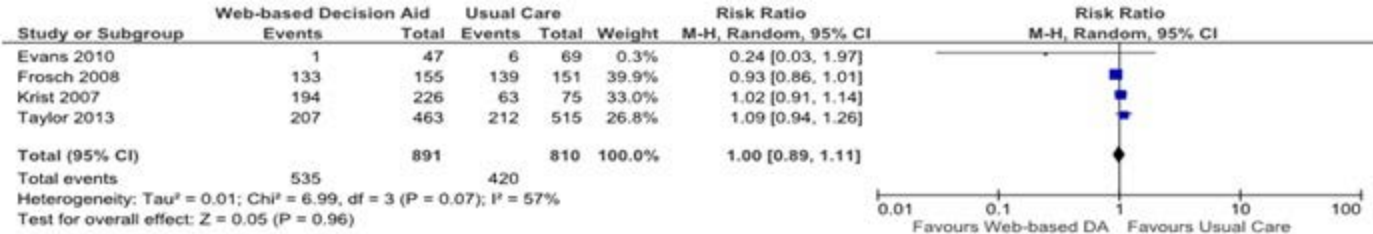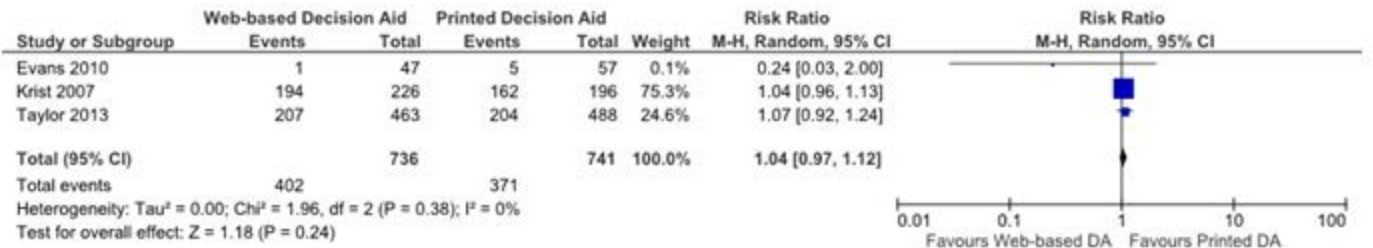

Supplement: Multimedia Appendix 6 [file jmir_v20i6e228_app6.pdf]
